# Screen-Printed Electrodes Modified with Metal Nanoparticles for Small Molecule Sensing

**DOI:** 10.3390/bios10020009

**Published:** 2020-02-01

**Authors:** Daniel Antuña-Jiménez, María Begoña González-García, David Hernández-Santos, Pablo Fanjul-Bolado

**Affiliations:** Metrohm DropSens S.L., Edificio CEEI-Parque Tecnológico de Asturias, 33428 Llanera, Spain; daniel.antuna@metrohm.com (D.A.-J.); begona.gonzalez@metrohm.com (M.B.G.-G.); david.hernandez@metrohm.com (D.H.-S.)

**Keywords:** metal nanoparticles, bimetallic alloys, metal oxides, screen printed electrode, ink-mixing, drop casting, electrodeposition, electrocatalysis, enzymatic sensor, enzyme-free sensor

## Abstract

Recent progress in the field of electroanalysis with metal nanoparticle (NP)-based screen-printed electrodes (SPEs) is discussed, focusing on the methods employed to perform the electrode surface functionalization, and the final application achieved with different types of metallic NPs. The ink mixing approach, electrochemical deposition, and drop casting are the usual methodologies used for SPEs’ modification purposes to obtain nanoparticulated sensing phases with suitable tailor-made functionalities. Among these, applications on inorganic and organic molecule sensing with several NPs of transition metals, bimetallic alloys, and metal oxides should be highlighted.

## 1. Introduction

Screen-printed electrodes are well-known suitable platforms for sensing devices’ development. Technology involving its preparation implies the use of many different substrates, such as ceramic, plastic, paper, or glass. In addition, the possibility of producing patterns of cells or electrodes with different architectures, such as single electrodes or conventional three-or-more electrode cells, interdigitated electrodes, flow cells, etc., affords a wide range of different applications. Its easy preparation and cost-effective production allows mass production of customized electrode configurations with devices made of different substrates, geometries, shapes, and sizes.

In addition to its principal advantage that is customable manufacturing, screen-printed electrodes (SPEs) also offer the possibility of producing tailor-made surfaces to achieve desirable applications for the detection of specific analytes in several fields, such as industry, clinical, or academic research.

**Figure 1:**
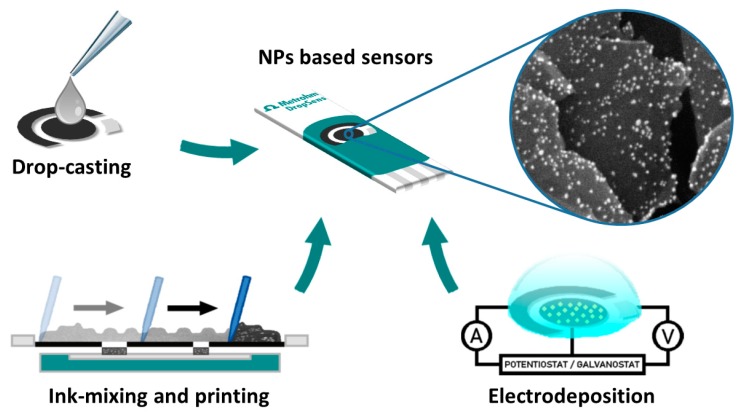
Schematic representation of the three main methodologies usually employed to modify SPEs with metal NPs.

Modification of the electrode surface in these devices is usually achieved by three well-known methods represented in Figure 1: Ink mixing with the modifying agent, electrochemical deposition of a metallic precursor, or drop casting of a preformed nanoparticulated material. The first method is carried out before ink curing and has more critical parameters, such as the curing temperature and mixing recipes, that need to be very well supervised in order to achieve batch reproducibility [1]. The other two methods are performed, after electrode preparation, on their surface, so they are more suitable for working with commercially available SPEs.

There is a vast literature on the use of metal NPs for the detection of heavy metal ions, and several reviews have been published [2,3,4,5,6]. So, the scope of this review was only centered on applications with the aim of detecting inorganic and organic molecules and the strategies employed to improve the analytical signal when using NPs as the sensing phase.

## 2. Modification Methodologies

### 2.1. Ink Mixing Method

The first approach used for screen-printed electrode modification with nanoparticles copies the usual methodology previously explored with modifiers onto carbon paste electrodes. Functionalization in these devices was achieved by mixing modifying material with the carbon-based paste that was subsequently pressed and polished.

In this way, ink mixing SPE modification consists in the preparation of an ink where three main components are always presented: Conductive particles usually made of carbonous material, a solvent/binder mixture that allows to transfer particulated matter onto the substrate, and the modifying agent, metal NPs in this case. Depending on the final application, the main parameters that should be optimized are the recipe of the ink, its rheology, substrate selection, and thermal curing.

Screen printing starts with the positioning of the printing media upon the mesh screen followed by the application of pressure with a squeegee that forces printing medium through the previously designed pattern. Finally, a curing temperature is applied to dry the ink. This procedure can be applied several times in order to obtain more layers of the material onto the substrate, either conductive ink or dielectric material. Further details of the procedure were reviewed elsewhere [7].

Being the first approach explored, original papers appear in the early 1990s. These publications employed conductive material attached directly to metal particles, so the modification consists of metallized carbon with platinum [8], palladium [9], or iridium [10,11] mixed with conventional inks used for screen printing to obtain second generation enzymatic sensors, where metallic particles are used as a catalyst. The main advantage of this methodology is in the fact that commercially available materials were simply mixed with printing binders and solvents, so only the mixing recipe was optimized.

Later, pristine metallic particles previously separated from carbon were mixed with conductive material and binder [12,13]. Although the percentage of metal dispersion can modulate the reactivity of the supported metal substrates, when the ink mixing approach is used, the analytical response is not equally affected. This behavior can be explained by the nature of the mixing process. As nanoparticulated material is mixed together, agglomeration occurs, diminishing the degree of dispersion, so, finally, bigger particles in the micro range are obtained with the same metal loading. This is the reason why only a few publications [14,15] are based on ink mixing with metal NPs, since the main advantage of NPs, a high surface area due to interparticle spacing and their nanometer size, is lost.

Recently, an interesting paper deals with suitable ink mixing modification procedures to achieve better results regarding the catalytic performance [14]. Due to the higher surface having more active sites, an inkjet-printing approach was used with capped silver NPs. The inkjet approach is based in the same principle, like screen printing, as an ink that is previously mixed is required. In this case, the prepared ink passes through a nozzle so th printing material is applied directly onto the surface and mild thermal treatment compared to heat curing is required. Moreover, environmentally friendly organic solvents like glycerol are employed, so the printing conditions are less aggressive.

Although this technique is more suitable for printing detailed patterns onto flexible substrates, inkjet printing affords thinner conductive substrates so more metallic particles are exposed to the analyte solution, diminishing the aggregation effect when compared to the ink mixing method. Moreover, inhibition is avoided due to the lack of paste additives used for ink mixing that can decrease the catalytic activity. The results obtained with this methodology with silver NPs show a greater catalytic response against hydrogen peroxide. One more improvement can be made by using a decapping method, where hydrochloric acid is used to dissolve the capped agent of silver NPs. Following this protocol, agglomerated particles can be interconnected, decreasing the resistance and affording better electron transfer and, consequently, improving the analytical performance [14]. This is the only approach, until now, that overcomes ink mixing drawback with metal NPs in a practical manner, but no further metals were tested.

### 2.2. Drop Casting Method

This is the easiest method employed to modify screen-printed electrodes. Only one parameter should be optimized: The final amount drop casted onto the electrode. This can be modulated by changing the size of the drop and the concentration of the metal NPs dispersed in the solution.

Sensors developed with this methodology are based in two main strategies: Direct modification of selected NPs onto the working electrode or ex-situ fabrication of composites made of NPs attached with carbonous nanomaterials.

The first strategy is easier to carry out, as no functionalization step is necessary. So, many metal NP solutions were used, such as bismuth [16], platinum [17,18,19], rhodium [20], gold [21], silver [22], copper [23], and nickel [24]. Sensors, thus prepared, are stable, even in flow injection systems, making easier and affordable sensing devices possible when compared to those obtained by the ink mixing method. On the other hand, agglomeration is the main drawback observed in these devices. As NPs were simply casted and dried onto the working electrode, NPs tends to aggregate while drying, so the final material attached to the surface has a microparticulated appearance, even when a nanosized material was casted initially.

In order to overcome this issue, the second strategy is more commonly used since more reproducible nano-sized metallic centers are obtained. NPs are synthesized in this way onto a conductive carbonous substrate, in a similar manner to the particulated metallized carbonous materials firstly employed with the ink mixing method. When compared to the successive casting in the two steps of carbon nanomaterial followed by NPs [21], these novel composites afford not only a “real” nanoparticulated substrate but also an increased electroactive area due to the nature of the carbon nanomaterials employed. Taking this advantage, platinum [25], silver [26], nickel [27], and gold [28] were used in combination with carbon nanotubes [25], nanoporous carbon [27], and reduced graphene oxide [26] or graphene oxide [28] to obtain novel composite sensing devices with improved catalytic performance. Recently, bimetallic clusters made of Cu-Ti, where porous titanium phosphate NPs were used as thee carrier for copper NPs, were also tested in a similar manner to carbonous materials, offering novel sensing platforms based only on metallic substrates [29]. All these published works are mainly focused on the previously synthesized composite than on the methodology itself, since the main novelty of these sensors is due to the use of a casted composite responsible for the improved catalysis.

### 2.3. Methods based on Electrochemical Deposition

This is the most common method used to modify SPEs with metal NPs because it is the best method to control the morphology of NPs in an accurate way. This methodology is based on the reduction of oxidized species, typically metallic water-soluble salts, at a fixed potential or current to obtain tailor-made metal particles grown on conductive substrates.

The parameters usually optimized are separated in two main groups: The ones related to the precursor solution where the salt type and concentration are involved, and the conditions of electrochemical deposition. A list of usual salts employed are AgNO_3_ [30,31,32,33,34] for silver NPs; HAuCl_4_ [31,35,36] and AuCl_3_ [37] for gold NPs; Bi(NO_3_)_3_ [16,38,39] for bismuth NPs; CoCl_2_ [40] for cobalt NPs; CuCl_2_ [41], CuSO_4_ [42], and CuNO_3_ [43,44] for copper NPs; NiCl_2_ [40,45] and NiSO_4_ [46,47] for nickel NPs; PdCl_2_ [48,49,50] for palladium NPs; H_2_PtCl_4_ [51], H_2_PtCl_6_ [30,52,53,54,55,56], and PtCl_2_ [37] for platinum NPs; RhCl_3_ [57] for rhodium NPs, etc. Although higher concentrations of precursor allow bigger particles to be obtained, the size and shape are usually controlled electrochemically; so, the precursor concentration usually tends to be high enough to have sufficient material susceptible to be deposited and it is not often optimized [33]. With this in mind, two parameters are crucial to control the size and shape of growth NPs: The potential or current applied and the time of deposition. The last one modulates the amount and size of metal onto the electrode in such a way that a longer time gives rise to higher amounts of NPs with a bigger particle size [37]. The first parameter will be discussed separately in subsequent sections since potential changes are the basis of potentiostatic techniques while current changes are the basis of galvanostatic techniques.

#### 2.3.1. Electrochemical Methods based on Potentiostatic Techniques

These methods are based on the application of a fixed potential. When a specific potential to deposit NPs is applied, their size and shape can be modulated. As the potential becomes more negative, the nucleation rate increases, so more NPs are obtained with a smaller particle size, increasing the electroactive surface area. Focusing on the shape, negative potentials around −0.1 V (vs. a silver pseudo-reference electrode) give rise to more spherical NPs since growth is favored more than nucleation while a more negative potential affords an heterogeneous morphology [53]. Generally, the sensitivity of sensors made by potentiostatic deposition increases as the deposition potential is more negative in combination with a higher deposition time. In this way, when applying a deposition potential equal or more negative than −0.5 V (vs. an Ag/AgCl pseudo-reference electrode), platinum NPs’ fast growth creates a heterogeneous concentration of platinum ions around NPs, making the formation of polyhedron shapes that grow up faster than the rest of the facets possible, forming flower-like nanostructures [51]. Similar structures are obtained with bismuth [38] at −1 V and gold [58] at −0.2 V vs. Ag/AgCl pseudo-reference electrodes.

Although morphology is important, “best photo” is not often the goal but improved analytical performance, so the optimization has to be carried out to achieve a better response depending on the application goal. For example, more negative potentials (−0.6 V vs. Ag/AgCl) and shorter deposition times (190 s) than the ones employed to achieve nanoflowers are employed for the deposition of platinum onto ultramicroelectrodes [52]. Mild reduction conditions are employed to obtain a wider linearity range and higher sensitivity for bromide detection with rhodium NPs [57] and for ascorbic acid detection with gold NPs [35].

Deposition is usually performed in one potentiostatic reduction step by applying a potential negative enough to afford a reduction of the metal species to the zero-valence state, but sometimes, two steps [32] or more [54] are employed. In these cases, the objective is to more precisely control both the nucleation and growth rates. NPs, thus prepared, show improved homogeneity and the possibility of having more accurate results.

Examples of a well-controlled electrodeposition process are bimetallic alloys. These systems are based on particles that combine two different metals in one single particle as a core shell [54] approach or simply by co-deposition [59]. The main goal of nanoparticulated alloys is the combination of the advantages from the different metals involved and the minimization of the disadvantages of each one [60]. A good example of the accuracy level that is needed when these NPs are fabricated is the nanoflower-like morphology obtained with the Pt-Pd system by using a multi-step potentiostatic protocol [54]. A mixture of platinum and palladium salts are reduced under an initial constant potential, provoking the formation of numerous crystal seeds. A subsequent multi-potential step electrodeposition repeated for 50 cycles promotes precise growth of the colloidal nanostructures onto preformed seeds. This protocol allows porous nanostructures with a large surface area and abundant catalytic sites against peroxide reduction to be obtained. The application of only one of the steps from the whole protocol affords particles with serious agglomeration that reduces the surface active sites and electrocatalytic properties dramatically, so a very well controlled growth has to be achived with these bialloy systems.

Electrodeposition protocol is not the only parameter that should be taken into account when bimetallic alloys are developed. The selection of metals deposited, and the order of deposition are also crucial to achieve the best results. For example, a hydrazine sensor prepared with core-shell Cu-Pd NPs shows better results when copper is deposited first [61].

Cyclic voltammetry is also employed for electrodeposition, but control is more difficult because more parameters need to be optimized, such as the scan rate, scan cycles, and potential window. Published works that use this technique with noble metals are mainly focused on the novelty of the substrates employed like graphite nanosheets [49], carbon nanotubes [55], fullerenes [48], graphene [46], and graphene oxide [59] than electrodeposition itself.

Bimetallic alloys were also obtained when using cyclic voltammetry as the electrochemical technique. Gold and silver have been deposited together onto the carbonous working electrode, improving the sensitivity by slightly giving up the dynamic range [59]. Silver as a substrate was also employed in another bimetallic system, where bismuth was electrodeposited onto silver SPE, creating an alloy capable of catalyzing hydrogen peroxide oxidation, but the mechanism involved remain unknown [39].

Cyclic voltammetry is a useful technique when copper as a metal is employed, since passivated copper with an oxide/hydroxide layer is the species responsible for the catalysis of sugars and amino acids. Since the catalytic properties of CuNPs towards these analytes depend on the size, shape, and nature of CuNPs, several studies have been carried out to clarify the complex mechanism of electrocatalysis [44,62]. The first approach consists of cyclic scans successively applied in several cycles to achieve the deposition of copper onto the SPE and also passivation of the reduced metal to obtain catalytic centers in a two-step protocol. This methodology affords well-controlled cubic copper NPs without the aid of protective agents [43] with good sensing properties. Copper nanobelt can be synthesized via the potentiostatic method by applying a high potential and longer time of deposition. When nanobelts are compared with NPs, the response against sugar increases dramatically due to a higher oxidized surface being obtained as a greater area is exposed to ambient self-oxidation [41].

Amino acids can also be detected with copper NPs because their carboxylic and amine terminals act like a chelating agent in a bidentate ligand. Their complexation with oxidized species of copper is capable of decreasing the detection potential at 0 V, while electrocatalysis increases this potential from the +0.4 to +0.8 V range. Interestingly, this phenomenon only occurs when 100-nm-sized copper NPs are electrodeposited onto SPEs, so accurate control of the particle size needs to be controlled. For this reason, a photo-irradiated electrodeposition method was developed based on potentiostatic electrodeposition applied under a xenon light source, because the modulation intensity of light is capable of controlling the size growth of copper NPs [44].

#### 2.3.2. Methods based on the Galvanostatic Technique

These methods apply a constant negative current capable of reducing precursor metallic salt. The more negative the current applied, the higher the nucleation rate achieved, similar to potentiostatic methods. The deposition time is also a crucial parameter.

Although this technique is not extensively used, the employment of current instead of potentials is more convenient when using SPEs [63]. Due to the pseudo-reference electrode, potentials can change when oxidizing media are used in the deposition step. As the morphology of the nanoparticle surface is responsible for the final analytical response of the sensor, small variations in the reference potential give rise to less accurate results. The application of a constant current minimizes the effects of pseudo-referenced systems [42] but good control of the current applied is still necessary. In this way, negative current densities (around −0.2 mA cm^−2^) afford homogeneous nanoflower-like structures when nickel is reduced [47] while a more negative current (around −1.8 mA cm^−2^) affords a more heterogeneous particle size distribution when copper is deposited [42]. This phenomenon observed with copper can be explained because nucleation and growth seem to take place at different times so two different particle size are obtained. Taking into account the deposition time, a suitable time window has to be applied when using sputtered paper as the substrate, as a larger time can produce gold detachment [64].

The galvanostatic technique also offers the possibility of synthesizing porous metallic substrates with a high surface area via hydrogen evolution-assisted electrodeposition. This methodology consists in the application of a very large current density (around 1.4 A cm^−2^) to the electrode system, provoking a quick reduction of metal ions at the working electrode of the SPE combined with the arising of numerous bubbles of hydrogen, hindering normal diffusion of the remaining ions of the precursor salt. Since no ion can occupy the space of hydrogen bubbles, the deposition is only achieved in the inner space among these bubbles so the final result is the formation of highly porous architectures where pores of hundreds of nanometers are easily obtained. This method has been employed to achieve 3D porous nickel structures with an extremely large electroactive area [45]. A greater surface area to increase the electroactivity can be obtained with subsequent electrodeposition. A combination of hydrogen evolution-assisted galvanostatic reduction followed by potentiostatic electrodeposition was assayed in a two-step protocol [36]. Firstly, a large current density affords a highly porous gold substrate, and secondly, several cyclic voltammetry cycles are applied to obtain conventional gold NPs deposit. The final electrode is made of microporous and nanoparticulated gold, offering a dramatic increase in the analytical signal.

Bimetallic alloys can also be obtained using galvanostatic methods to combine the advantages of different metals. Nickel and cobalt can be co-deposited together, providing similar current values for glucose detection than those obtained with cobalt NPs but at a lower applied potential [40].

Galvanic displacement was also explored for bimetallic alloys’ electrosynthesis [30]. It is based on the phenomenon observed when a moderately active metal is partially replaced (e.g., oxidized) by a less active or more noble metal. Based on this fact, silver NPs were deposited first to serve as the active metal. Secondly, SPE modified with silver NPs is immersed into platinum salt solution and left without the application of any potential or current. Platinum displaces silver on the surface so the final particle is made of a core of silver and a shell of platinum, with no electrochemical step applied. Platinum shell extension can be modulated by changing the time of contact. More time provides more shell growth and a better response against peroxide catalysis.

### 2.4. Other Methods for NPs Modification

Although the majority of SPEs modified with metallic NPs are obtained with the above-mentioned methods, there are others that have been used to fabricate these devices.

Chemical deposition has been assayed for nanostructurated sensing phase synthesis. Reducing precursor salt onto the SPE allows hydroxilated copper nanowires that can be oxidized via thermal treatment to be obtained, affording a high catalytic surface area of copper oxide suitable for glucose detection [65]. The main drawback is that the extension of the reduced material cannot be controlled with accuracy with this approach. Chemical deposition of iridium onto graphene was also tested as a pH sensor in a cheap and portable pHmeter [66].

In a more aggressive way, spark discharge was also applied onto SPEs. It is based on the application of an electric field high enough to create an ionized electrically conductive channel through air between two electrodes. Bismuth NPs were synthesized in this way by using a +1.2 kV DC power supply, where bismuth wire is connected to the positive pole and the working electrode is connected to the negative pole. Applying several electric discharge (sparking) cycles under atmospheric conditions, homogeneously distributed bismuth NPs of 2–5 nm separated and covered with a carbonous layer are produced in the surface of the working electrode. With this approach, it is possible to detect nanomolar concentrations of riboflavin without deaeration of the sample due to the presence of the carbon shell layer formed around the bismuth oxide NPs provoked by the own nature of the sparking process. The presence of this layer restricts oxygen interaction with bismuth NPs during voltammetric detection, affording lower blanks that allow measurements at lower concentrations [67].

## 3. Roles and Applications of Metal Nanoparticles

In this section, the main roles of the SPEs modified with NPs are revised. As mentioned above, heavy metal detection is the main application of metallic particles, but the extensive bibliography exceeds the scope of this review. Despite this, a brief mention is made of bismuth NPs that are the most commonly sensing phase employed in the detection of heavy metals since they are considered the most popular “green” substitutes for the classical mercury drop or film electrodes. Due to its wide cathodic range and its low toxicity, this allows the detection of heavy metal ions in a similar manner as mercury, but without the environmental risk of mercury waste disposal, such as bioaccumulation and acute toxicity by vapor inhalation [68,69]. Electrodes modified with bismuth can be achieved by the use of a precursor or simply by nanoparticle deposition using different modification methods [3].

In this section, the applications achieved with other metal NPS will be discussed, focusing on the specific role developed in the sensing device. In this way, three main different roles are considered. Metal NPs can act as a catalyst in enzymatic and non-enzymatic devices, as sensing phases for direct detection of several analytes, or as anchorage substrates for sensing platforms. In addition, two tables summarizing the real applications of SPEs based on metallic NPs (see Table 1) and bimetallic alloys (see Table 2) are included for clarification.

### 3.1. As catalyst in Enzymatic and Non-Enzymatic Devices

Metallic NPs are capable of catalyzin relevant processes, such as peroxide reduction or carbohydrates oxidation, making the detection of enzymatic products, sugars, and amino acids in real samples possible. The following discussion is focused on the catalytical effect of several NPs against these and other analytes.

#### 3.1.1. Hydrogen Peroxide Monitoring

Hydrogen peroxide monitoring is essential because it participates in classical enzymatic reactions extensively used in the biosensing field and it is an additive in many food, pharmaceutical, and environmental goods. Among enzymatic sensing devices, the most commonly used ones are based on peroxidases and oxidases due to their high selectivity and sensitivity. The reaction pathways of these enzymes involve hydrogen peroxide as the reagent or product, respectively, so it is the target analyte when detecting substrates with this two kind of enzymes. The monitoring is achieved directly in first generation biosensors, by the use of mediators in second generation biosensors or by the catalytic center of the enzyme in third generation biosensors. Although these successive improvements increase the use of enzymatic devices, they usually have poor chemical and long-term stability that limits their fabrication and increases the cost of the final device.

**Figure 2:**
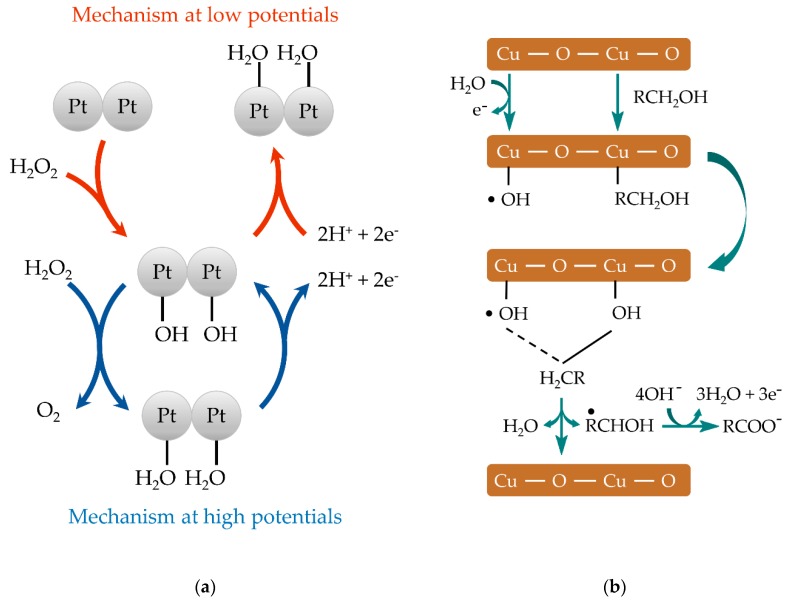
Schemes showing the proposed mechanism of (**a**) hydrogen peroxide oxidation onto platinum NPs [71] and (**b**) sugar oxidation onto copper NPs [62].

Platinum group metallic NPs are a well-known electrocatalyst towards the oxidation or reduction of hydrogen peroxide [70]. The mechanism involved [71] is shown in Figure 2a. Taking into account its catalytical properties, it was possible to develop disposable glucose biosensor made by platinized carbon particles [8]. Further studies based on platinum nanoflowers electrodeposited potentiostatically affords a sensor with a wide linear range capable of monitoring glucose in serum samples [51].

Similar to platinum, palladium shows high catalytic activity towards several electrochemical processes, making it possible to perform sensors based on screen-printing technologies [12]. Although being more expensive than platinum, oxygen reduction reaction onto palladium is only a slightly lower potential than that onto platinum, so it is possible to monitor dissolved oxygen with good reproducibility in ground and tap water [50]. As a peroxide catalyst, palladium NPs were employed for monitoring glucose with an SPE strip based on palladium-dispersed carbon ink [9].

Iridium NP-modified SPEs by the ink mixing approach were also used as transducers for enzymatic reactions. By means of peroxide detection, uric acid was detected via uricase reaction [10].

As noble metal NPs are capable of detecting peroxide at low overpotential when compared to bare classical electrodes, they were also employed in enzyme-free devices. There are many advantages in the electrochemical detection of peroxide without the presence of an enzyme, such as the improvement in stability and reproducibility and the possibility of obtaining simple and inexpensive devices [72]. Moreover, it is now possible to prepare noble metal NPs with a highly controllable size, shape, surface charge, and physicochemical characteristics for specific electrocatalytic applications [73,74,75]. Combining all these facts with other capabilities, such as low toxicity, high surface area, wide surface functionalization chemistry, and colloidal stability, these nanomaterials were extensively used in recent years to perform new biosensing platforms and devices by themselves.

Platinum is the most common noble metal employed for non-enzymatic sensors. Due to its good electrocatalytic activity towards peroxide, platinum can be used to detect this molecule in several real samples, such as cosmetics [53], household goods [30], or food and beverages [25], with good recoveries. A simple electrodeposition step is needed [37] to perform sensors with this method, affording a facile and robust methodology to monitor peroxide “in-situ” and making it even susceptible to academic demonstrations with hair lightener [56] and whitening strips [18] as real samples.

Like platinum, rhodium NPs were also used for peroxide detection. By a simple drop casting method, SPEs modified with rhodium NPs were used to detect hydrogen peroxide produced by autoxidation of polyphenols in tea extracts [20].

In a similar manner to platinum-like metals, other noble metals, such as gold, silver, and copper, are employed in sensing devices. Sensors developed with these NPs show less catalytic activity towards hydrogen peroxide and they are very dependent on the particle size and preparation steps but offer cheaper devices compared to platinum-modified SPEs.

Silver NP-based SPEs offer better catalytic properties towards peroxide detection than bulk silver electrodes. Unfortunately, sensors are only stable for a week because the main drawback of silver-based nanomaterials relies on its inherent and fast oxidation, so the stability is compromised for mass production purposes [14]. Drop casting methodologies were also applied in combination with carbon nanomaterials attached directly to silver NPs [26] or by the ink mixing method [22] to enhance the sensitivity.

Focusing on the field of biosensing and organic molecules, only a few works employing bismuth NPs were published [76] because the detection capabilities with enzymes or organic compounds were not comparable to those obtained with heavy metal stripping assays. Moreover, due to high background limitations, these electrodes need to be deaerated before using in many practical applications when very low detection limits are necessary.

Bismuth can act as a catalyst for hydrogen peroxide detection but only when it is combined with silver. Electrodeposition of bismuth NPs onto silver electrodes forms an alloy capable of catalyzing the reduction of hydrogen peroxide, including in real cosmetic samples. The existence of these alloys can open the door to a new metallic catalyst for sensing devices [39].

Metal oxide nanoparticles were extensively used for sensing gases [77], but the applications related to that field exceed the scope of this review. When focusing on electrochemical sensing and biosensing devices, not many applications were developed when employing screen printing technology, and much less in real samples [78]. Although transition metal oxides are sensitive to the same analytes as their reduced counterparts, and similar modification methods were employed, the preparation procedures do not allow nanoparticulated electrode surfaces to be obtained.

Acting as a catalyst for peroxide oxidation, metallic oxides can be classified depending on their nature. The first group corresponds to common oxides, such as CuO [65,79,80] and NiO [81], with MnO_2_ [82] as the most representative one, and the second group correspond to oxides of platinum group metals, such as RuO_2_ [83], RhO_2_ [84] and PtO_2_, PdO, or IrO_2_ [85]. The latter afford more expensive sensors but are more chemically stable. Electrodes modified with these compounds are prepared by ink mixing [86], the electrodeposition method [80,87], or ex-situ growth of copper with graphene and subsequent drop casting methodology [79]. Assays with real samples were only achieved in the past years in combination with enzymes for glucose monitoring purposes in fruits [83] and food [84].

Metal alloys prepared with several elements, such as Pt-Ag [30] and Pt-Pd [54], afford a better response towards peroxide [30] and glucose [54] than each metal separately. Good detection is achieved in real samples, such as antiseptic and laundry boosters [30].

#### 3.1.2. Carbohydrate Monitoring

Several metallic elements are capable of catalyzing the hydrolysis of carbohydrates, making the detection of these analytes in real samples possible. 

Copper-modified SPEs were evaluated for sugar detection. The proposed mechanism for sugars’ oxidation [62] onto copper NPs is schematized in Figure 2b. For this purpose, electrodeposition of copper nanospheres [42] or nanobelts [41] and chemically synthesized copper nanowires [65] have been prepared. Nickel NPs show good electrocatalytical properties towards sugar oxidation in a similar way. They have been applied in non-enzymatic devices against glucose in food [24,42,47], blood [45], or urine [46] as real samples. It is worth mentioning that a voltammetric or amperometric pretreatment must be previously done with these NPs to obtain metal oxide species, such as oxyhydroxides, which are responsible for the catalytic oxidation of carbohydrates.

Gold surfaces are also capable of catalyzing sugar oxidation due to the presence of chemisorpted hydroxyl anions forming hydrous gold oxides, which are believed to be the catalytic component of gold electrodes. The main applications of this behavior relies on the development of novel non-enzymatic sensing in the health science field. In this way, glycated hemoglobin can be monitored in serum with electrodeposited gold nano-flowers [58]. Glucose levels in serum and blood can also be analyzed with electrodeposited porous gold nanostructures [36] or drop-casted gold NPs onto graphene nanocomposites [28]. Sugars were also quantified in beverages with gold NPs electrodeposited onto a gold-sputtered paper, obtaining similar results to those obtained using a commercial enzymatic kit [64]. In addition, advanced devices were also recently tested, like an enzymatic fuel cell to monitor glucose and oxygen in human saliva [88].

Although metal oxides are capable of detecting hydroxide [81] directly and can be used in non-enzymatic sensing of carbohydrates via commercial oxide powder modification [62] or electrodeposition of nanoparticulated material [80], no real samples were assayed.

Bimetallic alloys based on NPs capable of catalyzing carbohydrate oxidation were prepared. Cu-Ti [29] and Ni-Co [40] offer an improved response towards glucose, combining the capabilities of both metals involved.

**Table 1:** Application of NPs with real samples.

NPs	Modification		Analyte	Detection		Performance		Sample	Year	Ref.
	Tech.	Parameters		Tech.	Parameters	Linear range	LOD			
**Ag**	DC	12 μL AgNP-rGO composite, RT	H_2_O_2_	AD	−0.3 V	0.5 μM to 12 mM	0.21 μM	Contact lens care solution	2016	[26]
	PE	CA, −1.2 V, 10 s	Sulfite	AD	+0.4 V	1.96 to 16.66 mM	1.99 mM	Beverages	2013	[31]
	PE	*Step 1*: CA, 0.13 V, 5 ms *Step 2*: CA, 0.24 V, 25 s	Metronidazole	DPV	E_amp_: −0.1 V 0.075 Vs^−1^	3.1 to 310 μM	0.4 μM	Serum, Urine, and Tablets	2012	[32]
	PE	CA, −1.2 V, 120 s	Lamotrigine	DPCSV (CA+DPV)	A: −0.90, 147 s	0.33 to 1.50 μM	0.372 μM	Pharmaceuticals	2007	[33]
	PE	CA, −1.2 V, 20 s	Chloride Bromide Iodide	LSV	−0.2 to 0.6 V, 0.01 V s^−1^	3 μM to 100 μM 5 μM to 90 μM 5 μM to 80 μM	3 μM 5 μM 5 μM	Synthetic sweat	2018	[34]
**Au**	IM	Ionophore based ink	Trazodone	OCP	-	10 μM to 10 mM	6.8 μM	Pharmaceuticals	2018	[15]
	DC	-	Carbofuran	DPCSV (CA+DPV)	A: 0 V, 60 s DPV: −0.2 to 0.35 V, E_p_: 0.15 V, t_p_: 0.3 s, E_step_: 0.01 V	1–250 µM	0.22 µM	Food	2017	[21]
	DC	1.8 µL AuNPs Graphene composite, RT, 12 h	H_2_O_2_ Glucose	AD	−0.2V	0.2 to 4.2 mM 2 to 10mM	− 180 µM	Blood	2010	[28]
	PE	CA, +0.18 V, 10 s	Sulfite	AD	+0.3 V	9.8 to 83.33 μM	9.79 μM	Beverages	2013	[31]
	PE	CA, +0.18 V, 50 s	Ascorbic acid	DPV	−0.2 to 0.8 V, 0.1 V s^−1^ E_p_: 0.012 V, t_p_: 0.07 s, E_step_: 0.025 V	1.9 to 16.6 μM	0.99 μM	Serum	2017	[35]
	HEA-GE	*Step 1*: CP, 3 Acm^−2^, 100 s, RT *Step 2*: CV, 10 cycles, −0.7 to 0.4 V, 0.05 Vs^−1^	Glucose	AD	−0.2 V	1.5 and 16 mM	25 µM	Serum	2018	[36]
	PE	CA, −0.2 V, 150 s	Glycated hemoglobin	CV	0 to −0.6 V, 0.1 Vs^−1^ Calibrated at −0.45 V	2 to 20%	0.65%	Serum	2019	[58]
	PE	CV, 5 cycles +0.4 to −0.6 V, 0.05 Vs^−1^	Sulfide	DPCSV (CA+DPV)	A, +0.4 V, 60 s DPV: +0.4 to −0.9 V, E_p_: 0.008 V, t_p_: 0.05 s, E_step_: 0.1 V	0.05 to 1.5 μM	0,2 uM	Tap water	2016	[59]
	GE	CP, −100 μA, 6000 s	Glucose	CV	−0.3 to +0.5 V, 0.1 Vs^−1^	0.01 to 5 mM	6 µM	Beverages	2017	[64]
**Bi**	PE	CA, −1 V, 4 min	Phenol	CA	+0.8 V, 150 s	5 to 100 µM	480 nM	Wastewater	2010	[38]
	PE	CV, 20 cycles −0.6 to 0.3 V	H_2_O_2_	CV	−0.3 to −1.3 V	100 µM to 5 mM	57 µM	Cosmetic	2011	[39]
	SD	1.2 kV, 20 cycles	Riboflavin	SWV	0 to −0.8 V, Freq: 50 Hz, E_amp_: 0.05 V, E_step_: 0.0015 V	1 to 100 nM	0.7 nM	Multivitamin	2015	[67]
**Cu**	GE	CP, −225 μA, 60 s	Glucose Fructose Arabinose Galactose Mannose Xylose	CA	+0.65 V, 100 s	1 μM to 10 mΜ	0.57 μM 0.61 μM 1.0 μM 0.89 μM 1.3 μM 1.04 μM	Honey and beverages	2017	[42]
	IM	Ink with 50% of Cu(OH)_2_ nanorods	Ascorbic acid	CA	0 V, 25 s	0.0125 to 10 mΜ	6 mM	Tablets Urine	2017	[89]
**Ir**	IM	Ink with 0,9:5 of Ir-C powder (5 % Ir)	Triglyceride	CA	+0.15 V, 30 s	Up to 10 mM	-	Serum	2008	[11]
**Ni**	DC	15 μL (10 g L^−1^) *Activation:* A, −1.5 V, 600 s in NaOH 0.1 M	Glucose Fructose Mix 1:1	AD, FIA	+0.7 V, 2 mL min^−1^	0.05 to 1 mM	0.06 mM 0.04 mM 0.04 mM	Honey	2012	[24]
	HEA-GE	CP, 0.1 A, 30 s	Glucose	CA	+0.5 V, 100 s	0.5 μM to 4 mM	0.07 μM	Blood	2013	[45]
	PE	CV, 40 cycles, 0.05 Vs^−1^, 0 to −1.5 V *Activation:* CV, 40 cycles, 0.1 Vs^−1^, 0 to +0.8 V in 0.1 M NaOH	Glucose	AD	+0.6 V	0.2 to 9 mM	4.1 μM	Urine	2013	[46]
	GE	CP, −25 μA, 60 s *Activation:* CV, 50 cycles, 0.1 Vs^−1^, +0.2 to +0.7 V in 0.1 M NaOH	Glucose Fructose	CA	+0.6 V, 120 s	25 to 1000 μM	Between 8 μM and 20 μM	Food	2016	[47]
**Pd**	PE	CV, 10 cycles, 0.05 Vs^−1^ −0.25 to +1.2 V	Dopamine	DPV	−0.1 to +0.6 V	0.35 to 135.35 µM	0.056 µM	Injection	2015	[48]
	PE	CV, 20 cycles, 0.02 Vs^−1^ +1.2 to −0.25 V	Hydrazine	AD	−0.05 V	0.05 to 1415 µM	4 nM	Drainage water	2016	[49]
	PE	CA, −0.6 V, 180 s	Dissolved O_2_	CV	0.5 to −0.3 V, 0.02 V s^−1^	Up to 250 µM	-	Ground and tap water	2006	[50]
**Pt**	DC	12 μL, RT, 24 h	H_2_O_2_	AD	−0.3 V	1 µM to 10 mM	0.43 µM	Contact lens care solution	2016	[17]
	DC	20 μL, dried at 80 ˚C, 10 min	H_2_O_2_	AD	0.345 V	Up to 0.1 mM	6.6 µM	Whitening Strips	2015	[18]
	DC	10 μL (2 g L^−1^), dried at 40 ˚C, 180 min	Ethanol	LSV	−1 to 1 V, 0.05 V s^−1^	15 to 102 mM	15 mM	Beverages	2017	[19]
	DC	0.5 µL PtNP-MWCNT composite, RT	H_2_O_2_	CA	+0.3 V, 60 s	10 to 100 µM	10 µM	Green tea	2018	[25]
	PE	CA, −0.5 V, 300 s	H_2_O_2_	CA	−0.7 V, 30 s	500 µM to 20 mM	32.8 µM	Serum	2017	[51]
	PE	CA, −0.4 V, 900 s	H_2_O_2_	AD	+0.7 V	6 to 215 µM	7.6 µM	Hair lightener Antiseptic Plant extract	2017	[53]
	PE	CA, 12.4 V, 12 min	H_2_O_2_	AD	+0.7 V	Up to 6.5 mM	80 µM	Hair lightener	2018	[56]
**Rh**	DC	15 μL, RT	H_2_O_2_	AD	0 V	5 to 600 μM	2 μM	Tea extracts	2015	[20]
	PE	CA, −0.25 V, 480 s	Bromide	CSV (CA+LSV)	A: +1.25 V, 20 s LSV: +1 to −0.25	Up to 40 mM	39 μM	Seawater Pharmaceuticals	2019	[57]

**AD**: Amperometric detection; **CA**: Chronoamperometry; **CP**: Chronopotentiommetry; **CV**: Cyclic Voltammetry; **CSV**: Cathodic Stripping Voltammetry; **DC**: Drop-Casting; **DPCSV**: Differential-Pulse Cathodic Stripping Voltammetry; **DPV**: Differential Pulse Voltammetry; **FIA**: Flow Injection Analysis; **GE**: Galvanostatic electrodeposition; **HEA-GE**: Hydrogen-Evolution-Assisted Galvanostatic Electrodeposition; **IM**: Ink-Mixing; **LSV**: Linear Sweep Voltammetry; **OCP**: Open Circuit Potential; **PE**: Potentiostatic electrodeposition; **RT**: Room Temperature; **SD**: Spark Discharge; **SWV**: Square Wave Voltammetry; **Tech.**: electrochemical technique.

**Table 2:** Application of bimetallic NPs with real samples.

NPs	Modification		Analyte	Detection		Performance		Sample	Year	Ref.
	Tech.	Parameters		Tech.	Parameters	Linear range	LOD			
**Cu-Ti**	DC	4 μL dried at RT	Glucose	CA	+0.5 V, 60 s	25 μM to 2 mM	7 μM	Honey Plasma	2017	[29]
**Pt-Ag**	PE-GD	*Step 1:* CA, −0.3 V, 900 s *Step 2:* 0.2 mM H_2_PtCl_6_ at pH 3.4 with 0.2 mM AA for 2.5 hours	H_2_O_2_	AD	+0.7 V	2.2 to 67 µM	0.34 µM	Antiseptic and Laundry boosters	2019	[30]
**Pt-Pd**	PE	*Step 1:* CP, 0.4 V, 20 s *Step 2:* 50 cycles of: CP, 0.5 V, 0.2 s CP, 0.4 V, 10 s	H_2_O_2_ Glucose	AD	−0.4 V	0.005 to 6 mM Up to 16 mM	0.87 μM 10 μM	Simulative blood	2012	[54]
**Au-Ag**	PE	CV, 5 cycles, +0.4 to −0.6 V 0.05 Vs^−1^	Sulfide	LSCSV (CA+LSV)	A, +0.2 V, 30 s LSV, +0.2 to −0.9 V 0.05 Vs^−1^	0.5 to 12.5 μM	0,2 μM	Water	2016	[59]
**Cu-Pd**	PE	*Step 1, Cu:* CA, −0.7 V, 300 s *Step 2, Pd:* CA, −0.6 V, 180 s	Hydrazine	AD, FIA	+0.2 V 0.5 mL min^−1^	2 to 100 µM	270 nM	Cigarette tobacco	2005	[61]

**AD**: Amperometric detection; **CA**: Chronoamperometry; **CP**: Chronopotentiommetry; **CV**: Cyclic Voltammetry; **DC**: Drop-Casting; **FIA**: Flow Injection Analysis; **GD**: Galvanic Displacemenet; **GE**: Galvanostatic electrodeposition; **LCSV**: Linear-Scan Cathodic Stripping Voltammetry; **LSV**: Linear Sweep Voltammetry; **PE**: Potentiostatic electrodeposition; **RT**: Room Temperature; **Tech.**: electrochemical technique.

### 3.2. As sensing Phase for Other Analytes

Metal NPs are also capable of monitoring other analytes, opening the path for new applications. Platinum NPs are capable of catalyzing the oxidation of small organic molecules, such as ethanol and formaldehyde, making the development of an alcohol sensor for wine and beer [19] and a gas sensor against formaldehyde possible [52].

Palladium nanoparticles are also capable of detecting formaldehyde and other analytes, such as hydrazine and sulphuric acid, with the classical approach of ink mixing. In addition, hydrogen was also tested in a proof-of-concept assay, making possible the application of palladium-based SPE sensors as gas detectors [12]. Although no real samples were assayed in this work, subsequent publications based on carbon nanomaterial substrates were successfully applied to monitor dopamine in pharmaceuticals [48] and hydrazine in wastewater samples [49]. Electrodeposition of rhodium NPs also gives rise to a good platform to detect bromide in seawater and pharmaceuticals [57].

Iridium NPs are also capable of monitoring NADH produced in the dehydrogenase oxidation of glycerol. With this approach, a triglyceride biosensor was developed with a good correlation in bovine and human serum [11]. In a similar way to other proton-responsive metals, iridium oxide NPs in combination with graphene were tested as pH sensor [66].

The affinity of sulphur against gold offers the possibility of performing direct detection of sulphur species, obtaining devices capable of detecting sulfite in beverages [31] and free sulfide in tap water [59]. Other molecules were also detected directly onto gold NPs or by mediation of the ion association complex [15], making the monitorization of ascorbic acid in serum [35], carbofuran in food [21], or trazodone in a potentiometric sensor stable up to 7 months [15].

Silver affinity towards sulfur and halide species was also explored for sensor development purposes. Sulfite was detected amperometrically with silver NPs in drinking water, pickle juice, and vinegar [31], and bromide, chloride, and iodide were detected voltammetricallly in synthetic sweat [34]. Focusing on organic molecules, direct detection with electrodeposited silver NPs is possible against metronidazole [32] and lamotrigine [33] in pharmaceutical tablets.

Direct detection with bismuth NPs was only achieved when using deposition onto SPEs by sparking discharge. With this approach, it is possible to detect riboflavin in a nanomolar concentration in multivitamin real samples [67].

Copper NPs are capable of a complex amino acid as previously mentioned, so α-,β- [44] and γ- amino acids [43] can be monitored in flow systems with good correlation and detectability. Although the potential employed for the detection of amino acid can be decreased dramatically when using copper NPs via complexation, no real samples were assayed in these reports. Other analyte, such as ascorbic acid, was tested with copper NPs via drop-casting methodology [23], but again, no real sample was assayed. Only by using hydroxilated copper nanorods with an ink-mixing approach can ascorbic acid be detected in urine with good recoveries [89].

Alloys of Cu-Pd [61] and Au-Ag [59] were developed to increase the sensibility for hydrazine [61] and free sulfide [59] detection in real samples of tap water [59] and cigarettes [61].

Metal oxides were successfully tested for direct sensing of pharmaceuticals in real samples by using other metal-transition oxides not previously mentioned like ZrO alone [90] or doped with rare earth metals [91].

### 3.3. As platforms for Sensing Phases

Metallic NPs can also serve as carriers for sensing phases in other applications not mentioned before.

Gold NPs were extensively used as an anchoring platform. Easy functionalization and tailor-made size, shape, and nature are advantages that offer a wide range of applications in the electrochemical sensors field [92]. A recent review deals with different approaches and novelties published until now with gold-based sensors for the detection of small molecules, DNA, and aptamers [93]. Another advantage in the use of gold NPs consists in the strong binding of thiol ligands onto their surface due to the soft character of both gold and sulfur. Monolayers onto gold NPs were extensively used to stabilize them and also for functionalization purposes in many different applications [94].

Bismuth NPs were also explored as a biosensing platform in mediator-free enzymatic SPE devices. These NPs are capable of complex oxidase activity tissues, affording a substrate capable of detecting phenolic compounds, thus direct monitoring the product of the enzymatic reaction instead of hydrogen peroxide was achieved. Deposition of cationic bismuth and negatively charged mushroom tissue together offers immobilization of polyphenol oxidase enzyme onto carbon SPEs, making the determination of phenol possible [38]. With a similar approach, the same group developed a tyrosinase-based biosensor against phenol and cathecol [16].

Only a few more metallic NPs were studied as platforms for sensing devices with a rich related bibliography. Iron and cobalt NPs are one of the most assayed ones due to its low-cost production, tailor-made magnetic properties, easy functionalization processes, and biocompatibility. Iron NPs are often used as a magnetic carrier more than a catalyst [95] while cobalt NPs are mostly employed to achieve inks for electronic 3D printing [96], so the applications and SPE-based devices achieved exceed the scope of this review.

Bimetallic clusters can also serve for biotin labelling detection in multivitamin tablets with Cd-Ti functionalized with neutravidin [97] and SERS enhancement effect to study 4-mercaptopyridine adsorption-desorption onto electrodeposited dendritic Au-Ag NPs [98].

## 4. Conclusions

After practically 25 years from the first publication, metal NPs are still nowadays employed as modifying agents in sensing devices based on screen printing technology. Ink mixing was the first approach explored capable of offering metallized SPE for sensing development. The main drawbacks of this method is the agglomeration of NPs, complex ink recipes, and bad reproducibility among batches. Drop casting methodologies offer a suitable approach to modify SPEs since the modification is carried out after ink preparation. NPs casted onto working electrodes offer a high active surface against analyte but agglomeration is also achieved. Nanoparticulated material with accurate tailor-made sizes and shapes is only obtained when using electrodeposition. Potentiostactic techniques are employed more due to their use for years in the classical three-electrode cell with a well-controlled reference electrode. However, galvanostatic techniques are more appropriate when working with SPEs. Deposition under a controlled current is not affected by potential variations from the pseudo-reference electrode, offering similar capabilities to control the nucleation and growth of metallic NPs as potentiostatic techniques. Large-scale synthesis is the main drawback of this method due to electrodeposition having to be done with each sensor separately, and the deposition step can be a large time-consuming process when thinking in large batch preparation.

Recent scientific publications dealing with these sensing phases are focused on the development of novel strategies capable of affording a better analytical performance based on an increasing surface area or novel modifying agent synthesis. Newly developed electrochemical techniques that can increase the electroactive area in a great amount are based on previous knowledge not applied before with SPEs like hydrogen-evolution-assisted galvanostatic electrodeposition and spark discharge, or the use of carbonous nanomaterials. On the other hand, bimetallic clusters as modifying agents can be electrodeposited with an accurate growth control, affording novel surfaces with advantages from several metals in one particle, offering unknown metal synergies suitable for future screen printing devices based on metal NPs.
